# Supplementary material for: COVID-19 and Female Genital Mutilation/Cutting and child marriage: An online multi-country cross sectional survey
Source: PLoS One. 2024 Oct 31;19(10):e0304671. doi: 10.1371/journal.pone.0304671 (PMC11527327; doi:10.1371/journal.pone.0304671)

# **Supporting information file 1**

**S1 Fig.** Perception of additional of risk female circumcision due to COVID-19 on girls (N=278)


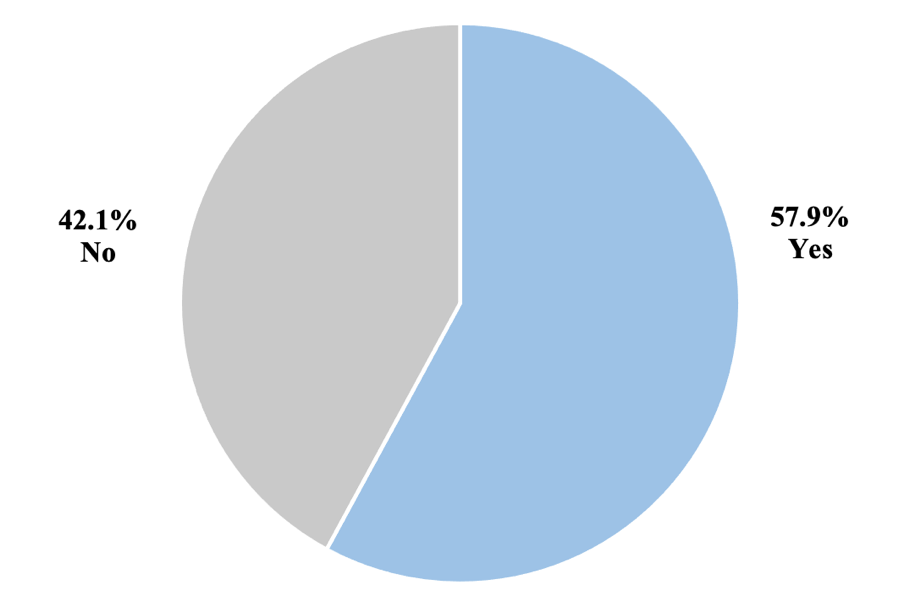

Supplement: S1 Fig — This chart shows the proportion of participants who felt the pandemic had increased the risk of FGM/C. (DOCX) [file pone.0304671.s001.docx]
