# Supplementary material for: COVID-19 and Female Genital Mutilation/Cutting and child marriage: An online multi-country cross sectional survey
Source: PLoS One. 2024 Oct 31;19(10):e0304671. doi: 10.1371/journal.pone.0304671 (PMC11527327; doi:10.1371/journal.pone.0304671)

**Supporting information File 2**

**S2 Fig.** Perception of additional risk of childhood marriage due to COVID-19 on girls (N=1,284)


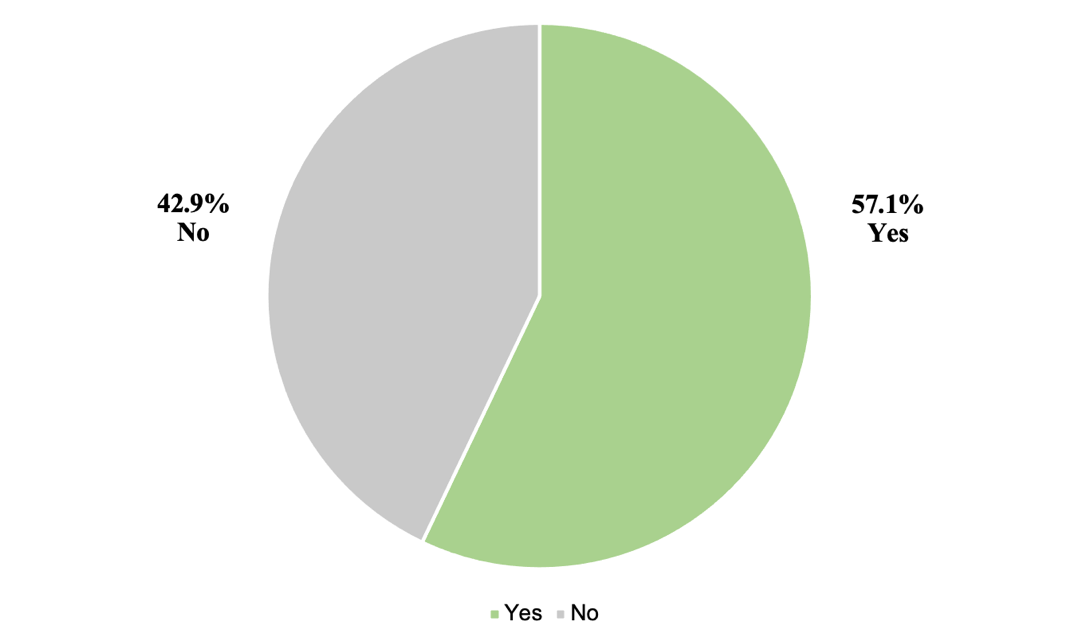

Supplement: S2 Fig — This chart shows the proportion of participants who felt the pandemic had increased the risk of child marriage. (DOCX) [file pone.0304671.s002.docx]
