# Supplementary material for: COVID-19 and Female Genital Mutilation/Cutting and child marriage: An online multi-country cross sectional survey
Source: PLoS One. 2024 Oct 31;19(10):e0304671. doi: 10.1371/journal.pone.0304671 (PMC11527327; doi:10.1371/journal.pone.0304671)
Supplement: S1 Table — This table shows the survey items relating to FGM/C and child marriage and the response options for each question. (DOCX) [file pone.0304671.s003.docx]

**Supporting Information File 3**

**S1 Table.** Survey Items Relating to FGM/C and Child Marriage in the I-SHARE survey, 2020-2021

| Survey Questions | Response Options |
| --- | --- |
| FGM/C | |
| 1. Is female circumcision practised in your community? | - No - Yes - I Don't Know - NA |
| 1. Do you have a daughter who is at the age that circumcision is generally done? | - No - Yes - NA |
| 1. Before the COVID-19 social distancing measures, did you intend to circumcise your daughter? | - No - Yes - NA |
| 1. Did the COVID-19 situation change your plans to circumcise your daughter? | - No - Yes, I decided to do the circumcision later than planned - Yes, I decided to do the circumcision sooner than planned - Yes, I decided to cancel the circumcision |
| 1. In general, do you feel that because of COVID-19 girls are at a higher risk of circumcision? | - No - Yes, somewhat higher risk - Yes, much higher risk - I don't know - NA |
| Child Marriage | |
| 1. Does early marriage (marriage before the age of 18 years) happen in your community? | - No - Yes - I Don't Know - NA |
| 1. Do you have a child between the age of 10 and 18 years old? | - No - Yes, one - Yes, more than one - NA |
| 1. Before the COVID-19 social distancing measures, did you intend to arrange a marriage for your child(ren) that are between 10 and 18 years old? | - No - Yes - NA |
| 1. Did the COVID-19 situation change your plans to arrange a marriage for your adolescent child(ren)? | - No - Yes, I have cancelled the marriage plans - Yes, I will arrange the marriage later than planned - Yes, I will arrange the marriage sooner than planned |
| 1. In general, do you feel that because of COVID-19 girls and boys are at a higher risk of early marriage? | - No - Yes, somewhat higher risk - Yes, much higher risk - I don't know - NA |
