# Supplementary material for: COVID-19 and Female Genital Mutilation/Cutting and child marriage: An online multi-country cross sectional survey
Source: PLoS One. 2024 Oct 31;19(10):e0304671. doi: 10.1371/journal.pone.0304671 (PMC11527327; doi:10.1371/journal.pone.0304671)
Supplement: Attachment — Submitted filename: Response tto Reveiwers.docx [file pone.0304671.s005.docx]

Reviewer's Responses to Questions

**Comments to the Author**

1. If the authors have adequately addressed your comments raised in a previous round of review and you feel that this manuscript is now acceptable for publication, you may indicate that here to bypass the “Comments to the Author” section, enter your conflict of interest statement in the “Confidential to Editor” section, and submit your "Accept" recommendation.

Reviewer #2: All comments have been addressed

Response: Thank you

2. Is the manuscript technically sound, and do the data support the conclusions?

The manuscript must describe a technically sound piece of scientific research with data that supports the conclusions. Experiments must have been conducted rigorously, with appropriate controls, replication, and sample sizes. The conclusions must be drawn appropriately based on the data presented.

Reviewer #2: Partly

Response: Thank you

3. Has the statistical analysis been performed appropriately and rigorously?

Reviewer #2: Yes

Response: Thank you

4. Have the authors made all data underlying the findings in their manuscript fully available?

The [PLOS Data policy](http://url.uk.m.mimecastprotect.com/s/HrbTCr914S3Ow7Wu4l0wa?domain=plosone.org) requires authors to make all data underlying the findings described in their manuscript fully available without restriction, with rare exception (please refer to the Data Availability Statement in the manuscript PDF file). The data should be provided as part of the manuscript or its supporting information, or deposited to a public repository. For example, in addition to summary statistics, the data points behind means, medians and variance measures should be available. If there are restrictions on publicly sharing data—e.g. participant privacy or use of data from a third party—those must be specified.

Reviewer #2: Yes

Response: Thank you

5. Is the manuscript presented in an intelligible fashion and written in standard English?

PLOS ONE does not copyedit accepted manuscripts, so the language in submitted articles must be clear, correct, and unambiguous. Any typographical or grammatical errors should be corrected at revision, so please note any specific errors here.

Reviewer #2: Yes

Response: Thank you

6. Review Comments to the Author

Please use the space provided to explain your answers to the questions above. You may also include additional comments for the author, including concerns about dual publication, research ethics, or publication ethics. (Please upload your review as an attachment if it exceeds 20,000 characters)

Reviewer #2: Overall the appropriate changes were made, the limitations of the study are clear and well explained.

It would be helpful to explain that the FMG C and child marriage were optional in the survey and that the survey did not include questions about the type of COVID restrictions.

**Response:** We have added this clarification in the methods section under the paragraph on survey items. The las two sentences now reads as follows: “Note that the survey items for FGM/C and child marriage were included as optional components of the main survey and 14 countries included these two components in the survey. The survey did not include questions about the type of COVID restrictions”.

7. PLOS authors have the option to publish the peer review history of their article ([what does this mean?](https://url.uk.m.mimecastprotect.com/s/XSMACvZ14t0ZOqRCAm3MM?domain=journals.plos.org)). If published, this will include your full peer review and any attached files.


If you choose “no”, your identity will remain anonymous but your review may still be made public.


**Do you want your identity to be public for this peer review?** For information about this choice, including consent withdrawal, please see our [Privacy Policy](https://url.uk.m.mimecastprotect.com/s/TCVUCwr14SZn0k2c1-z-4?domain=plos.org).

Reviewer #2: **Yes:** Mercedes Alarcon
